# A survey of sports and rehabilitation professionals on foam rolling applications, contraindications, and adverse events - does the science reach professionals’ perceptions?

**DOI:** 10.3389/fphys.2025.1659081

**Published:** 2025-09-03

**Authors:** Katja Bartsch, Gunda Slomka, Christian Baumgart, Christina Offermann, Robert Schleip, Jürgen Freiwald, Werner Klingler, Christoph Egner, Jan Wilke, Matthias W. Hoppe

**Affiliations:** ^1^ Associate Professorship of Conservative and Rehabilitative Orthopaedics, TUM School of Medicine and Health, Technical University Munich, Munich, Germany; ^2^ Institute of Sport Science, University of Hildesheim, Hildesheim, Germany; ^3^ Department of Movement and Training Science, University of Wuppertal, Wuppertal, Germany; ^4^ Department of Medical Professions, Diploma University of Applied Sciences, Bad SoodenAllen, Germany; ^5^ Department of Anaesthesiology, SRH Hospital Sigmaringen, Sigmaringen, Germany; ^6^ Department of Experimental Anaesthesiology, Ulm University, Ulm, Germany; ^7^ Department of Movement Science, Institute of Sports Science, University of Klagenfurt, Klagenfurt, Austria; ^8^ Department of Neuromotorics and Movement, University of Bayreuth, Bayreuth, Germany; ^9^ Department of Exercise Science, Faculty of Educational Sciences, Institute of Sport Science and Motology, Philipps University of Marburg, Marburg, Germany

**Keywords:** self-myofascial release, fascia, massage, recovery, treatment

## Abstract

Foam Rolling (FR) is a type of self-massage used in sports and rehabilitation. The evidence on its effects has significantly increased in recent years; however, the extent to which novel research findings on FR have been implemented in practice remains unknown. Thus, we aimed to survey and document responses on the application, contraindications, and adverse events of FR among German-speaking sports and rehabilitation professionals. A cross-sectional online survey was conducted, which included 21 items relating to four key topics: (i) participant characteristics, (ii) FR application parameters, (iii) contraindications, and (iv) adverse events. Overall, 776 participants consented to participate. 68.6% of respondents indicated using FR in their practice. Average duration of FR was recommended at 3.2 min per body region. Smooth rollers were primarily used (82.9%). Pursued goals and observed effects were largely congruent and subjective methods were mainly used to assess effects. 90.9% of FR-users considered contraindications in practice, with pain being the most commonly noted factor. Headaches and tissue inflammation were the most reported side effects. While 32.4% believed that no adverse events can be expected, most respondents found however adverse events such as tissue inflammation plausible. An assumed lack of scientific evidence for positive effects was the most frequently cited reason for non-use of FR. Although our results demonstrate congruency between research findings and practical application across various parameters, researchers and practitioners should collaborate closely to disseminate knowledge, establish guidelines, and expand the FR evidence base, particularly regarding adverse events.

## 1 Introduction

Over the past decade, Foam Rolling (FR) has gained popularity in sports science and rehabilitation. Accompanying this trend, the available evidence has grown substantially: While the mechanisms of FR are not yet fully understood (Behm and Wilke, 2019; Wiewelhove et al., 2019), its effects have been analyzed in recent systematic reviews and meta-analyses (Duarte França et al., 2024). In this context, FR yields positive short-term effects regarding an increased range of motion (ROM) (Cheatham et al., 2015; Grieve et al., 2022; Konrad et al., 2021; Martínez-Aranda et al., 2024; Skinner et al., 2020; Wilke et al., 2020) and reduced pain perception (Beardsley and Škarabot, 2015; Hendricks et al., 2020; Wiewelhove et al., 2019). Also, it does not negatively affect athletic performance (e.g., strength performance) (Beardsley and Škarabot, 2015; Cheatham et al., 2015; Konrad et al., 2022). However, in some instances, minor performance improvements (e.g., sprinting) have been noted (Martínez-Aranda et al., 2024; Wiewelhove et al., 2019). The absence of adverse effects on athletic performance is seen as positive, particularly concerning the use of FR prior to sports (Hendricks et al., 2020; Skinner et al., 2020). Noteworthy is that there is no consensus regarding the optimal application parameters of FR such as duration, frequency, or type of roller to date (Dȩbski et al., 2019; Wiewelhove, 2021).

Insights into how sports and rehabilitation professionals, such as athletic trainers or physiotherapists, implement FR in their practice are limited. Only one study has examined the connection between scientific evidence on FR and its practical application, investigating the goals, observed effects, and application parameters through a cross-sectional survey (Cheatham and Stull, 2018). A search in the PubMed database using the term “foam rolling” shows that most FR-related studies have been published since this survey^1^. Thus, further research is needed to assess how much the accumulated research has influenced practice.

Research on risk factors and adverse events associated with FR is scarce. Our group has previously conducted an international Delphi study addressing potential contraindications (conditions that make FR inadvisable) and cautions (factors that increase the risk of a serious short- or long-term adverse reaction or necessitate consideration for medical screening) related to FR (Bartsch et al., 2021). To the best of our knowledge, this is currently the only available evidence on risk factors of FR, aside from commentary and opinion pieces (Cheatham and Stull, 2018; Freiwald et al., 2016). Moreover, scientifically investigated case reports on adverse events (harmful incidents occurring during or after FR) have not been published yet. As a result, no study has assessed how possible contraindications, cautions, and adverse events of FR are factored into sports and rehabilitation practices to date.

Combining science and practice–specifically creating interactions between researchers and professionals in sports and rehabilitation–can enhance the effectiveness and safety of FR interventions. In addition, understanding how practitioners apply FR in their daily work can guide researchers when designing future studies and developing FR recommendations (Cheatham et al., 2018). Therefore, qualitative methods, such as online surveys, are widely accepted to bridge the gap between research and practice in the sports environment (Harper and McCunn, 2017).

Thus, this study aimed to survey health and sports professionals and document their responses regarding applying FR, considering contraindications, and assessing their experiences and possible adverse events.

## 2 Materials and methods

### 2.1 Participants

This cross-sectional online survey recruited German-speaking sports and rehabilitation professionals from Germany, Austria, and Switzerland. The study included sports and rehabilitation professionals with qualifications such as sports scientists, therapists (e.g., physiotherapists, occupational therapists), and trainers/coaches. The survey invitation and link to the electronic questionnaire were distributed through various methods: email invitations, social media posts (e.g., Facebook, Instagram, Twitter), blog posts from educational institutions, and announcements made during faculty lectures. The survey period lasted 64 days.

### 2.2 Research design

We conducted a descriptive cross-sectional survey. The questionnaire was developed by an interdisciplinary group of eleven researchers, including researchers from sports science, medicine, physical therapy, applied health and therapeutic sciences, and human biology. Ten of the researchers had previously published peer-review paper(s) on foam rolling and thus contributed subject-relevant expertise to the development of the questionnaire. The initial structure and items of the questionnaire were developed based on the results of a literature review of PubMed indexed publications. The literature review revealed the study conducted by Cheatham, Stull and Amber-Wright (2018) to be the sole previously published cross-sectional survey on the application of foam rolling among sport or rehabilitation professionals, which was therefore used as starting point for the development of the questionnaire. The survey was further devised over multiple discussion rounds among the interdisciplinary researcher group, taking the current state of research into account. Iterative translation was performed by three panel members and subsequently discussed with the entire panel to ensure linguistic and cultural accuracy. Back-translation into English language was performed and checked for congruency. The final survey consisted of 21 questions, addressing four key topics: (i) participant characteristics, (ii) application parameters of FR, (iii) contraindications of FR, and (iv) adverse events of FR. Participant data included age, gender, professional qualification, working years in practice, work setting, and educational training related to FR practices. For application parameters, the context of FR application, used devices, recommended duration and reasonable frequency of FR, goals pursued with FR, observed effects, and types of measurements utilized to evaluate the effects achieved by FR were assessed.

Regarding contraindications, it was inquired if sports and rehabilitation professionals considered potential contraindications of FR and which ones they took into account. For adverse events, it was documented which were observed by sports and rehabilitation professionals and what long-term consequences they deemed conceivable. Some categorical questions permitted participants to select multiple responses (professional qualification and work setting, reasons for non-use of FR, context, goals, observed and measured effects of FR application, and conceivable long-term adverse effects of FR). Alongside closed questions, some items allowed open-ended responses (reasons for non-use of FR, contraindications considered in practice) (Harper and McCunn, 2017).

During the survey development, the preliminary questionnaire underwent two rounds of pilot testing with seven participants to determine feasibility and establish face validity. Based on feedback, revisions were made, and a final survey version was confirmed. The online survey was administered via SociSurvey^2^.

Participants gave their electronic informed consent before being directed to the survey questions. The survey was answered anonymously. Ethical approval was granted by the Ethics Committee of the DIPLOMA Hochschule, University of Applied Sciences (identification code EB 1027/2022), in line with the principles of the Declaration of Helsinki.

### 2.3 Data analysis

For questions with categorical responses, frequencies and percentages were calculated. The mean ± standard deviation (SD) was computed for continuous variables. For answers to open-ended questions (reasons for non-use and contraindications considered in practice), an inductive content analysis was performed. After the organization of raw data, themes were established. The process was repeated until data saturation was achieved. A hybrid approach of inductive and deductive content analysis was performed for the open question on considered contraindications. After two rounds of the inductive content analysis approach described above, data were sorted according to the International Classification of Diseases 11th edition (ICD-11). Data cleaning was performed for three items: For one participant, who indicated an age of 4 years, age was coded as missing value and not considered for data analysis. Regarding average FR duration in minutes per body region per session, missing values were coded and excluded from analysis for seven answers indicating a duration of 100 min or longer (answers in raw data: 2,000,000 min; 2,000 min, 3 × 500 min, 300 min, 150 min, 2 × 100 min). Frequency per week was coded as missing value and not taken into account for data analysis for two answers (311 x weekly; 315 x weekly). Statistical analysis was performed using SPSS version 29 (IBM SPSS, Armonk, NY, United States).

## 3 Results

### 3.1 Participant demographics

A total of 776 individuals gave consent to participate. Table 1 provides a summary of their characteristics. 694 participants provided information about their professional qualification(s), with 46.1% (n = 320) trainers/coaches, 40.1% (n = 278) therapists, and 21.3% (n = 148) sports scientists. Additionally, 14.3% (n = 99) participants indicated other qualifications such as physical education teacher, medical doctor, or yoga teacher. Work experience was reported with an average duration of 14.9 ± 11.3 years (N = 694; N indicates the total number of participants responding to a question, whereas n indicates the number of answers for subcategories of a question). Health sport was the most mentioned work setting (40.1%; n = 283), followed by non-organized (37.4%; n = 264), and organized recreational sport (28.5%; n = 201). Other work settings included physiotherapy clinics (24.8%; n = 175), hospitals and rehabilitation centers (14.2%; n = 100), competitive adult (14.2%; n = 100) and youth (7.2%; n = 51) sports, elite sports (6.2%; n = 44), and physical education (school) (4.5%; n = 32). 685 participants stated their education status related to FR, with 34.6% (n = 237) having completed an educational training course on FR.

**TABLE 1:** Participant demographics.

Participant demographics	
Age	Mean (±SD)
*Number of respondents*	*565*
Age in years	41.8 (±13.0)
Work experience	Mean (±SD)
*Number of respondents*	*694*
Working years in practice	14.9 (±11.3)
	Frequency (percentage of respondents)
Gender	
*Number of respondents*	*558*
Female	370 (65.5%)
Male	186 (32.9%)
Diverse	2 (0.4%)
Professional qualification(s)^a^	
*Number of respondents*	*694*
Trainer/coach	320 (46.1%)
Therapist	278 (40.1%)
Sports scientist	148 (21.3%)
Other	99 (14.3%)
Work setting(s)^a^	
*Number of respondents*	*706*
Health sports	283 (40.1%)
Non-organized recreational sports	264 (37.4%)
Organized recreational sports	201 (28.5%)
Physiotherapy clinic	175 (24.8%)
Hospitals and rehabilitation clinics	100 (14.2%)
Competitive sports	100 (14.2%)
Youth competitive sports	51 (7.2%)
Elite sports	44 (6.2%)
Physical education (school)	32 (4.5%)
Other	138 (19.5%)
Training course on FR	Mean (±SD)
*Number of respondents*	*685*
No	448 (65.4%)
Yes	237 (34.6%)

^a^ Respondents chose all options that applied to them; FR, foam rolling.

### 3.2 Application of foam rolling

Of the 685 participants, 68.6% (n = 470) indicated that they use FR in practice, while 31.4% (n = 215) do not. Within the group of FR-users, 439 gave information about the context in which they applied FR. Responses included individual training (i.e., person is practicing FR alone, e.g., as physical therapy homework) (64.9%; n = 285), personal training (i.e., one therapist/trainer/teacher guiding one individual practicing FR) (54.7%; n = 240), and group training (i.e., one therapist/trainer/teacher guiding several individuals practicing FR) (51.3%; n = 225).

Smooth surface rollers were most commonly used among the 439 FR-users (82.9%; n = 364), followed by rollers with surface structure (10.0%; n = 44) and rollers featuring a vibration function (1.8%; n = 8). Other used rollers included soft rollers, such as Pilates rolls or rollers with a recess for the spine.

The average recommended duration of FR per body region per session was reported at 3.2 ± 3.1 min (N = 429). The estimate for reasonable frequency per week was reported at an average of 3.1 ± 2.6 times weekly (N = 409).

**FIGURE 1:**
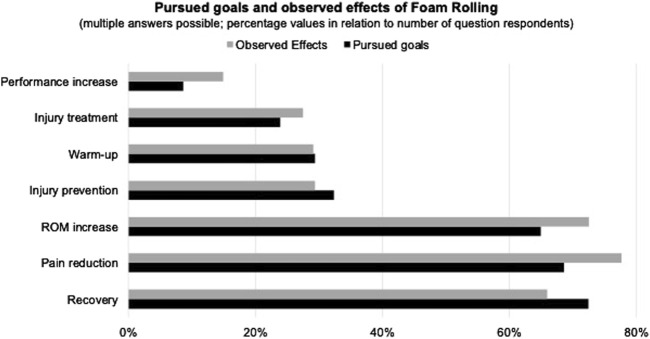
Relationship between pursued goals and observed effects of Foam Rolling. The graph illustrates the alignment between intended goals and actual observed effects of FR for respondents who gave answers to both survey items. Bubble sizes represent the percentage of total participants (n = 429). Notes: Multiple mentioned goals and effects were allowed; FR, Foam Rolling; ROM, range of motion.

**FIGURE 2:**
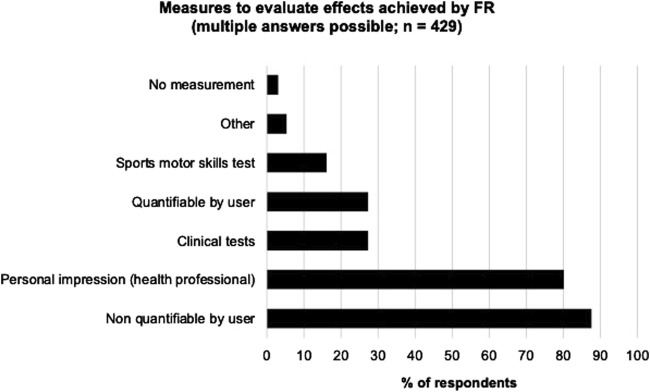
Measures used to evaluate effects achieved by Foam Rolling.

The most frequently mentioned goals for FR were recovery (72.4%; n = 318), pain reduction (68.6%; n = 301), and ROM increase (64.9%; n = 285). Among the 429 individuals, who reported observed effects of FR, the most frequently reported effects were pain reduction (77.6%; n = 333), ROM increase (72.5%; n = 311), and recovery (66.0%; n = 283). Figure 1 depicts pursued goals and observed effects of FR. When asking for the measures which were used to assess the effects of FR (N = 429), the majority of interviewed professionals answered non-quantifiable feedback from users (87.6%; n = 376), followed by personal impression (80.2%; n = 344), and clinical tests (27.3%; n = 117). Figure 2 gives an overview of the measures used to evaluate the effects achieved by FR.

Among the 215 non-users of FR, 192 gave a reason for non-use. The most frequently stated reason was an assumed lack of scientific evidence for its positive effects (32.3%; n = 62), followed by a lack of practicability in their work context (31.3%; n = 60) and not being acquainted with FR (29.7%; n = 57). Answers retrieved from the open-question format revealed further rationales for non-use of FR. Table 2 gives a summary of FR’s application parameters.

**TABLE 2:** Application parameters of FR.

Application parameters of FR	
Duration	Mean (±SD)
*Number of respondents*	*429*
Minutes per body region per session	3.2 (±3.1)
Frequency	Mean (±SD)
*Number of respondents*	*409*
Frequency of FR per week	3.1 (±2.6)
	Frequency (percentage of respondents)
Application of FR in practice	
*Number of respondents*	*685*
Yes	470 (68.6%)
No	215 (31.4%)
Context(s) of FR application^a^	
*Number of respondents*	*439*
Individual training	285 (64.9%)
Personal training	240 (54.7%)
Group training	225 (51.3%)
Other	28 (6.4%)
Type of Foam Roll primarily used	
*Number of respondents*	*439*
FR with smooth surface	364 (82.9%)
FR with surface structure	44 (10.0%)
FR with vibration function	8 (1.8%)
Other	23 (5.2%)
Goals for FR application^a^	
*Number of respondents*	*439*
Recovery	318 (72.4%)
Pain reduction	301 (68.6%)
Range of motion increase	285 (64.9%)
Injury prevention	142 (32.3%)
Warm-up	129 (29.4%)
Injury treatment	105 (23.9%)
Performance increase	38 (8.7%)
Other	30 (6.8%)
Observed effects of FR application^a^	Mean (±SD)
*Number of respondents*	*429*
Pain reduction	333 (77.6%)
Range of motion increase	311 (72.5%)
Recovery	283 (66.0%)
Injury prevention	126 (29.4%)
Warm-up before exercise	125 (29.1%)
Injury treatment	118 (27.5%)
Performance increase	64 (14.9%)
Other	43 (10.0%)
Measures used to assess effects of FR	
*Number of respondents*	*429*
Non-quantifiable feedback from user	376 (87.6%)
Personal impression	344 (80.2%)
Clinical tests	117 (27.3%)
Quantifiable feedback from user	117 (27.3%)
Sport motor skills testing	69 (16.1%)
Other	23 (5.4%)
Reasons for non-use^a^	
*Number of respondents*	*192*
Lack of scientific evidence for positive effects	62 (32.3%)
Lack of practicability in work context	60 (31.3%)
Not acquainted with FR	57 (29.7%)
Lack of equipment	28 (14.6%)
Other	49 (25.2%)
Uncertainty regarding contraindications	14 (7.3%)
Lack of suitability for target group	7 (3.6%)
Doubt about positive effects	7 (3.6%)
Pain during application	5 (2.6%)

^a^ Respondents chose all options that applied to them; FR: foam rolling.

### 3.3 Contraindications

90.9% (n = 389) of respondents considered contraindications in FR application, while 9.1% (n = 39) did not. The most frequently cited contraindications were pain (32.9%; n = 128), followed by varicose veins with (23.7%; n = 92), and acute inflammation (20.3%; n = 79). Table 3 provides a summary.

**TABLE 3:** Contraindications of FR.

Contraindications of FR	Frequency (percentage of respondents)
Considerations of contraindications in practice	
*Number of respondents*	*428*
Yes	389 (90.9%)
No	39 (9.1%)
Considered contraindications	
*Number of respondents*	389
Pain	128 (32.9%)
Varicose veins	92 (23.7%)
Acute inflammation	79 (20.3%)
Osteoporosis	75 (19.3%)
Acute injury	63 (16.2%)
Open wounds	62 (15.9%)
Vascular disease	54 (13.9%)
Acute fractures	49 (12.6%)
Thrombosis	42 (10.8%)

FR: Foam Rolling.

### 3.4 Adverse events

The surveyed sports and rehabilitation professionals assumed a relation between FR application and the occurrence of hematoma (180 mentions with an observed percentage greater than 0%), headache (157 mentions), and tissue inflammation (64 mentions) as most frequent potential side effects that occurred in a certain percentage of the end-users that were under their care. Considering conceivable adverse events of FR–regardless of whether the events occurred–tissue inflammation such as bursitis (37.9%; n = 145), desensitization of free nerve endings (26.4%; n = 101), and hardening of venous valves (18.0%; n = 86) were most frequently mentioned; 32.4% (n = 124) respondents indicated that no adverse events are to be expected with long-term application of FR (Table 4).

**TABLE 4:** Adverse events resulting from FR.

Adverse events (AE) of FR						
	Percentage of persons under care experiencing AE					
	0%	20%	40%	60%	80%	100%
Observed adverse events as result of FR						
Headache	220	117	32	4	4	0
Tissue inflammation (e.g., bursitis, tendinosis)	313	47	12	4	1	0
Rib fracture	356	17	4	0	1	0
Vertebral fracture (spinous process, transverse process)	369	7	1	0	0	1
Muscle fiber tear (immediately following FR application)	358	13	5	0	2	0
Hematoma	199	145	18	15	2	0
Other	114	26	4	5	1	3
Frequency (percentage of respondents)						
Conceivable adverse events^a^						
*Number of respondents*	383					
None	124 (32.4%)					
Tissue inflammation (e.g., bursitis, tendinopathy)	145 (37.9%)					
Desensitization of free nerve endings	101 (26.4%)					
Hardening of venous valves (possible reflux)	86 (22.5%)					
Local fibrosis	85 (22.2%)					
Hardening of arterial and venous vessels	69 (18.0%)					
Other	48 (12.5%)					

^a^ Respondents chose all options that applied to them; FR: foam rolling.

## 4 Discussion

Our online survey investigated the application of FR among sports and rehabilitation practitioners such as strength and conditioning coaches, fitness and personal trainers, and physiotherapists. The body of research on FR has considerably increased after the first online survey on roller massage use, which was carried out by Cheatham, Stull, and Ambler-Wright in 2018. Thus, our study provides valuable insight into how far the culminated FR research has reached practice. Furthermore, this work represents the first online survey documenting sports and rehabilitation professionals’ responses on contraindications and adverse events in a large sample of 776 participants.

Several main findings emerged from our study. First, FR appears to have multiple applications: Reported work contexts for FR use included various sports settings (e.g., health, recreational, competitive, and elite sports) and therapeutic environments (e.g., physiotherapy and rehabilitation clinics as well as hospitals). It may thus be hypothesized that FR can be a versatile tool in both performance- and health-related settings within sports and rehabilitation practices. Related target groups could benefit from the positive adaptations resulting from the mechanical stimulation of tissues initiated by FR.

The duration considered optimal by respondents in our survey slightly exceeds the protocols typically used in studies examining the acute effects of FR. On average, respondents indicated that they recommended FR for 3.2 min per body region per session. While no definitive dose-response relationship of FR on various outcome parameters has been established in the literature yet, standard durations for FR in research protocols range from 30 to 120 s per body region, applied for one to three times per session (Dȩbski et al., 2019; Michalak et al., 2024), and our findings support these durations. Concerning pain and muscle soreness reduction, studies include however longer durations of up to 600 s per body region; a minimum recommended dose of 90 s per muscle group, without an upper limit, has been documented also (Hughes and Ramer, 2019). For improvements in flexibility, various sources suggest durations between 30 and 120 s per body region to achieve optimal results (Behm et al., 2020; Hendricks et al., 2020). Since no upper limits have been identified in the literature, slightly longer rolling durations in practice seem reasonable, especially given that application in the field is often less standardized than in laboratory settings and may necessitate more thorough instruction and familiarization during execution. Participants reported an average frequency of 3.1 times per week as a reasonable rate. This finding aligns with studies on long-term effects (i.e., intervention durations of at least 4 weeks), where the frequency of FR application varies from twice a week to daily use (Pagaduan et al., 2022).

The most commonly used type of foam roll, featuring a smooth surface (82.9%; n = 364), aligns with the device primarily utilized in research; smooth surface rollers may also be the most available and affordable in practice. The second most used devices were foam rollers with surface structures (10.0%; n = 44). While research on the various surface properties affecting FR outcomes remains limited, initial study results indicate that grid and multilevel surface rollers may produce greater immediate post-intervention effects on ROM and perceived pain (Cheatham and Stull, 2019). This could be attributed to the increased pressure and isolated contact area achieved with rollers featuring surface structure, which can lead to greater mechanical deformation of the underlying tissue and, in turn, larger mechanical and neurophysiological effects (Cheatham and Stull, 2019; Curran et al., 2008). Conversely, more recent studies found no differences in perceived delayed onset of muscle soreness for varying textures and hardness levels of foam rollers (Michalak et al., 2024). Foam rollers with vibration functionality were the preferred tool for 1.8% (n = 8) of our study sample. Vibration foam rollers have gained increasing attention in both practice and research. In terms of ROM increases, various studies suggest that vibration foam rollers do not yield superior effects compared to traditional foam rollers (Kasahara et al., 2022; 2024; Nakamura et al., 2022; Wilke et al., 2020). In contrast to these findings, a meta-analysis revealed that vibration foam rollers led to greater ROM improvements in the hip and knee joints compared to conventional foam rollers without vibration (Park et al., 2021). Regarding performance and recovery, a further meta-analysis determined that vibration foam rollers are an effective tool for enhancing jump performance and recovery (Alonso-Calvete et al., 2022). Like conventional foam rollers, the dose-response relationships and protocol specifications for vibration foam rollers still need clarification in future research.

Regarding the goals for the FR application established by participants, the study results reflect the positive effects highlighted in the research presented in the introduction. Notably, the effects reported by respondents appeared to be aligned with the intended goals of the FR (Figure 1). However, most sports and rehabilitation professionals, who incorporated FR into their practices, indicated that they relied on non-quantifiable feedback from FR users and/or their personal impressions for evaluations. Worth to mention is that objective measures to assess the effects of FR, such as motor skills tests or clinical evaluations, were seldom employed in practice. This suggests a potential confirmation bias and reflects a gap in evidence-based practice among the sports and rehabilitation practitioners surveyed, which may also arise from a self-selection bias of those favorably inclined toward FR in our study sample (Andrade, 2020). Cognitive biases that distort assessments to support preferred hypotheses or beliefs have received increasing attention in therapeutic contexts (Blumenthal-Barby and Krieger, 2015; Born, 2024; Perna and Nemeroff, 2021) and in sport science (Beato et al., 2025). In the context of sports, the choice of measurement methods is critical in the evaluation of athletes’ development and potential. Confirmation bias can impact the selection of assessment methods, skew interpretation of test results, and thus negatively influence decision-making processes, which can in turn limit athletes’ progress and performance optimization (Beato et al., 2025). To ensure precision and evidence-based practice, various strategies have been identified to mitigate the impact and occurrence of confirmation bias (Beato et al., 2025; Born, 2024). To address confirmation bias in practice, employing objective, quantifiable alongside subjective measures can reduce the impact of confirmation bias when evaluating training and therapy outcomes (Born, 2024). Creating awareness and education around confirmation bias and evidence-based practices, fostering critical thinking skills and reflective practices, as well as encouraging open dialogue may furthermore mitigate the emergence of confirmation bias (Beato et al., 2025) and should therefore be included in the training and continuing education of sports and rehabilitation professionals. In addition, future research should concentrate on developing and refining protocols and user-friendly tools for training and therapeutic practice to assess the effects of FR.

Among respondents, who did not use FR in their practice, the most frequently cited reason for non-use was the assumed lack of scientific evidence for its positive effects (32.2%; n = 62). The evidence base surrounding FR has been continually expanding, with over 20 PubMed-indexed systematic reviews and meta-analyses published in the last 5 years, the majority of which focus on the positive effects of the intervention, such as ROM increases without negatively affecting athletic performance, or in some cases even positively impacting performance, as laid out before. Around a third of non-users were not aware of such positive effects; among users, only 8.7% (n = 38) referred to performance increases as goals for FR application. These responses may reflect ongoing challenges in translating evidence-based knowledge about FR into clinical routines, specifically the effective implementation of new knowledge and scientific evidence into practice (Warneke et al., 2024; Zidarov et al., 2013). Engaging coaches and practitioners to gather their perceptions and opinions, as this study has done, can serve as a critical step in bridging the gap between research and practice (Bishop, 2008). Future research could advance this approach further by utilizing methods grounded in implementation and participatory research to enhance knowledge translation (Prince et al., 2023).

The majority of users in our study (90.9%; n = 389) reported considering assumed contraindications for FR in practice. Research on FR contraindications is notably limited. Our previously published results from an international Delphi study with academic experts identified open wounds and bone fractures as potentially relevant conditions for which FR is inadvisable. Additionally, local tissue inflammation, deep vein thrombosis, osteomyelitis, and myositis ossificans were noted as cautions that increase the risk of adverse events or require further medical screening (Bartsch et al., 2021). In the present study, pain and varicose veins were the most frequently mentioned contraindications used, followed by acute inflammation, osteoporosis, acute injury, open wounds, vascular disease, acute fractures, and thrombosis (Table 3). While pain did not emerge as contraindication or precaution in our own previous Delphi study, it played a role in recent work concerning a related intervention: Severe pain felt by the patient has recently been identified as a precaution for instrument-assisted soft-tissue mobilization in an international Delphi study, albeit with weak agreement from the international experts (Cheatham et al., 2025). Pain has further been discussed as a contraindication for therapeutic massage (Batavia, 2004). Even though it can be argued that instrument-assisted soft-tissue mobilization and therapeutic massage share some commonalities with FR regarding their application characteristics (i.e., the application of mechanical loads such as compression or shear) and mechanisms of action (e.g., mechanotransduction), it can be assumed that differences in intensity are to be expected–for example, due to the difference in hardness and size of the tools used. Therefore, future research should shed light on dose-response relationships of these different techniques as they relate to precautions and contraindications such as pain. Furthermore, as pain is a complex phenomenon with multifactorial etiology, diverse features, comorbidities, and consequences (Kent et al., 2017; Loeser and Melzack, 1999), additional research is needed to evaluate the impact and associated risks of mechanical stimulation along all relevant characteristics. Varicose veins were the second most frequently mentioned contraindication in the present study. Like pain, they were not identified as precaution or contraindications in our own previous Delphi study. It is of note that various forms of medical compression therapy (such as compression stockings) are used in the non-invasive treatment of venous and lymphatic diseases (Rabe et al., 2020). It can therefore be concluded that compression loads on vascular structures are not necessarily disadvantageous. Individual studies on FR further suggest that compression loads during FR can positively impact vascular function (Okamoto et al., 2014). However, various undesirable events, including damage to vessels and nerves, have also been reported in connection with compression therapies. In this context, it has been recommended to avoid high local compression loads and to use soft materials in order to prevent harmful side effects (Rabe et al., 2020). This suggests that the effect of compression loads–and thus possibly also of FR–on the vascular and lymphatic system could be dose-dependent. A differentiated evaluation of possible dose-response relationships requires further research into the (pathophysiological) mechanisms of FR, taking into account various factors such as size and hardness of the used tool (Bartsch, 2024). The remaining considered contraindications show some overlap with the responses from our previous Delphi study. At the same time, it is not surprising that open wounds and acute fractures were not listed most frequently as contraindications in this study; these conditions may be so obvious that trainers and therapists rarely consider FR in these cases, leading to their minor role in our survey on FR contraindications; alternatively, these conditions might be rare in respondents’ practice, and therefore not commonly encountered. Regarding potential adverse events, it is noteworthy that 32.4% (n = 124) of respondents indicated that they expect no adverse events from the long-term application of FR. Thus, close to a third of the surveyed sports and rehabilitation professionals view FR as a safe intervention. This observation points to a knowledge gap regarding potential risks and reflects the scarcity of reported case studies on adverse events related to FR in the literature. Underreporting of adverse events, by both health professionals and patients, has gained attention in other medical fields, such as related to adverse drug reactions (Costa et al., 2023; García-Abeijon et al., 2023; Vural et al., 2015). Attitudes and lack of knowledge regarding the reporting of adverse events, which have been identified among the factors associated with underreporting, should be addressed in research and practice. Our own efforts to increase awareness about intervention vigilance (mirroring efforts to increase pharmacovigilance) include providing a reporting form for adverse events (in German language)^3^, which has also been shared with the participants of the present study. Conversely, a larger portion of our cohort deemed long-term adverse events (e.g., tissue inflammation, desensitization of free nerve endings; Table 4) plausible, underscoring the necessity for further research.

Even though our study provides an in-depth analysis of the application of FR, including its contraindications and adverse events, some limitations must be addressed, particularly regarding our recruitment strategy and survey mode. First, the study utilized convenience sampling as the invitation to participate in the online study was disseminated through various channels, such as email, different social media applications, as well as in-person announcements during faculty lectures. Consequently, the population from which we are drawing as well as response rate of our survey cannot be adequately quantified. Second, the study applied an online-format survey, which bears risk for under-coverage of specific population groups, for example, groups with less access to the internet (Bethlehem, 2010; Hsia et al., 2020), or whose internet usage patterns may have led to under-representation in the study. Third, respondents with biases may have self-selected into our sample. Accordingly, particularly those with a strong interest in or opinion about the study topic may have chosen to participate in the study; in addition, previous research has found personality type, number of previously taken surveys, and ethnic background to be associated with self-selection biases in online surveys (Bethlehem, 2010; Stone et al., 2024), all of which may also have played a role in our study. Fourth, our study was conducted exclusively with German-speaking professionals, thus limiting extrapolation of our findings to other geographical or cultural contexts. Taken together, lack of response rate, under-coverage of population groups, self-selection bias, as well as linguistic and cultural limitations limit the generalizability of our results. However, for the first time, our sample includes a wide range of professional qualifications, work settings, and FR application contexts, indicating a diverse and comprehensive representation within the field of sports and rehabilitation professionals. Therefore, our results can still provide valuable insights for various occupational groups. Furthermore, our results firstly reflect the participants’ perceptions and awareness of FR contraindications and adverse events. Future studies should assess these aspects with additional input from medical professionals to better understand the mechanistic foundations of FR. Additionally, clinical data are required to evaluate the risks associated with FR application. Such data may also help establish dose-response relationships of FR stimuli on acute and long-term positive or negative outcomes to define protocols and guidelines for FR application. It is furthermore noteworthy that 65.5% (n = 370) of participants identified their gender as female. Due to our recruitment and survey strategy, it is impossible to determine whether (i) gender preferences concerning FR application exist among sports and rehabilitation professionals in general, (ii) the recruitment strategy reached more women than men, and/or (iii) female sports and rehabilitation professionals are more likely to share their views on the subject of FR in an online survey. Future research should aim to clarify the extent of gender differences concerning FR.

## 5 Conclusion

Our study among a large cohort of sports and rehabilitation professionals examined the application of FR, assessed contraindications, and observed and identified adverse events. Regarding the duration, frequency, type of FR, and application goals, it is concluded that research-based findings and practical applications are mainly consistent. However, the positive effects of FR have not fully permeated practice. Additionally, research and practice should collaborate closely to establish practice protocols, share knowledge and guidelines about objective measurement methods for evaluating FR effects, and build evidence around FR’s contraindications and adverse events.

## Data Availability

The raw data supporting the conclusions of this article will be made available by the authors, without undue reservation.

## Footnotes

1 Searching the database PubMed for the search term „foam rolling” until the year 2024 yields 305 results (25 thereof categorized as systematic reviews); 240 of these research items have been published from the year 2019 onwards (23 systematic reviews, respectively).

2
www.socisurvey.de

3 See www.fasciaresearch.de

## References

[B1] Alonso-Calvete A. Lorenzo-Martínez M. Padrón-Cabo A. Pérez-Ferreirós A. Kalén A. Abelairas-Gómez C. (2022). Does vibration foam roller influence performance and recovery? A systematic review and meta-analysis. Sports Med. - Open 8 (1), 32. 10.1186/S40798-022-00421-2 35244802 PMC8897534

[B2] Andrade C. (2020). The limitations of online surveys. Indian J. Psychol. Med. 42 (6), 575–576. 10.1177/0253717620957496 33354086 PMC7735245

[B3] Bartsch K. (2024). Faszien in Therapie und Training: risikofaktoren für Foam Rolling und Untersuchungsmethoden zur Bewertung mechanischer Gewebeeingenschaften. 10.25593/OPEN-FAU-1314

[B4] Bartsch K. Baumgart C. Freiwald J. Wilke J. Slomka G. Turnhöfer S. (2021). Expert consensus on the contraindications and cautions of foam Rolling-An international Delphi Study. J. Clin. Med. 10 (22), 5360. 10.3390/JCM10225360 34830642 PMC8622134

[B5] Batavia M. (2004). Contraindications for therapeutic massage: do sources agree? J. Bodyw. Mov. Ther. 8 (1), 48–57. 10.1016/S1360-8592(03)00084-6

[B6] Beardsley C. Škarabot J. (2015). Effects of self-myofascial release: a systematic review. J. Bodyw. Mov. Ther. 19 (4), 747–758. 10.1016/j.jbmt.2015.08.007 26592233

[B7] Beato M. Latinjak A. T. Bertollo M. Boullosa D. (2025). Confirmation bias in sport science: understanding and mitigating its impact. Int. J. Sports Physiology Perform. 1 (aop), 1–6. 10.1123/IJSPP.2024-0381 39919722

[B8] Behm D. G. Wilke J. (2019). Do self-myofascial release devices release myofascia? rolling mechanisms: a narrative review. Sports Med. 49 (8), 1173–1181. 10.1007/S40279-019-01149-Y 31256353

[B9] Behm D. G. Alizadeh S. Hadjizadeh Anvar S. Mahmoud M. M. I. Ramsay E. Hanlon C. (2020). Foam rolling prescription: a clinical commentary. J. Strength Cond. Res. 34 (11), 3301–3308. 10.1519/JSC.0000000000003765 33105383

[B10] Bethlehem J. (2010). Selection bias in web surveys. Int. Stat. Rev. 78 (2), 161–188. 10.1111/J.1751-5823.2010.00112.X

[B11] Bishop D. (2008). An applied research model for the sport sciences. Sports Med. Auckl. N.Z. 38 (3), 253–263. 10.2165/00007256-200838030-00005 18278985

[B12] Blumenthal-Barby J. S. Krieger H. (2015). Cognitive biases and heuristics in medical decision making: a critical review using a systematic search strategy. Med. Decis. Mak. An Int. J. Soc. Med. Decis. Mak. 35 (4), 539–557. 10.1177/0272989X14547740 25145577

[B13] Born R. T. (2024). Stop fooling yourself! (diagnosing and treating confirmation bias). ENeuro 11 (10), ENEURO.0415–24.2024. 10.1523/ENEURO.0415-24.2024 39438140 PMC11495861

[B14] Cheatham S. W. Stull K. R. (2018). Roller massage: a commentary on Clinical Standards and Survey of physical therapy professionals - part 1. Int. J. Sports Phys. Ther. 13 (4), 763–772. 10.26603/ijspt20180763 30140569 PMC6088127

[B15] Cheatham S. W. Stull K. R. (2019). Roller massage: Comparison of three different surface type pattern foam rollers on passive knee range of motion and pain perception. J. Bodyw. Mov. Ther. 23 (3), 555–560. 10.1016/j.jbmt.2019.05.002 31563369

[B16] Cheatham S. W. Kolber M. J. Cain M. Lee M. (2015). The effects of self-myofascial release using a foam roll or roller massager on joint range of motion, muscle recovery, and performance: a systematic review. Int. J. Sports Phys. Ther. 10 (6), 827–838. 26618062 PMC4637917

[B17] Cheatham S. W. Stull K. R. Ambler-Wright T. (2018). Roller Massage: survey of physical therapy professionals and a commentary on clinical standards - part II. Int. J. Sports Phys. Ther. 13 (5), 920–930. 10.26603/ijspt20180920 30276024 PMC6159493

[B18] Cheatham S. W. Baker R. T. Loghmani M. T. Schleip R. (2025). International expert consensus on instrument-assisted soft-tissue mobilization precautions and contraindications: a modified Delphi Study. Healthc. Switz. 13 (6), 642. 10.3390/healthcare13060642 40150492 PMC11941819

[B19] Costa C. Abeijon P. Rodrigues D. A. Figueiras A. Herdeiro M. T. Torre C. (2023). Factors associated with underreporting of adverse drug reactions by patients: a systematic review. Int. J. Clin. Pharm. 45 (6), 1349–1358. 10.1007/s11096-023-01592-y 37247159 PMC10682061

[B20] Curran P. F. Fiore R. D. Crisco J. J. (2008). A comparison of the pressure exerted on soft tissue by 2 myofascial rollers. J. Sport Rehabilitation 17 (4), 432–442. 10.1123/jsr.17.4.432 19160916

[B21] Dȩbski P. Białas E. Gnat R. (2019). The parameters of foam rolling, self-myofascial release treatment: a review of the literature. Biomed. Hum. Kinet. 11 (1), 36–46. 10.2478/BHK-2019-0005

[B22] Duarte França M. E. Botti M. dos S. A. Ide F. C. Sinhorim L. Santos G. M. (2024). Effect of myofascial release techniques on internal biomechanics and their resultant application to sports: a systematic review. J. Bodyw. Mov. Ther. 40, 525–533. 10.1016/J.JBMT.2024.05.003 39593637

[B23] Freiwald J. Baumgart C. Kühnemann M. Hoppe M. W. (2016). Foam-rolling in sport and therapy – potential benefits and risks: part 2 – positive and adverse effects on athletic performance. Sports Orthop. Traumatology, 32(3), 267–275. 10.1016/j.orthtr.2016.07.002

[B24] García-Abeijon P. Costa C. Taracido M. Herdeiro M. T. Torre C. Figueiras A. (2023). Factors associated with underreporting of adverse drug reactions by health care professionals: a systematic review update. Drug Saf. 46 (7), 625–636. 10.1007/S40264-023-01302-7 37277678 PMC10279571

[B25] Grieve R. Byrne B. Clements C. Davies L.-J. Durrant E. Kitchen O. (2022). The effects of foam rolling on ankle dorsiflexion range of motion in healthy adults: a systematic literature review. J. Bodyw. Mov. Ther. 30, 53–59. 10.1016/j.jbmt.2022.01.006 35500979

[B26] Harper L. D. McCunn R. (2017). “Hand in glove”: using qualitative methods to connect research and practice. Int. J. Sports Physiology Perform. 12 (7), 990–993. 10.1123/IJSPP.2017-0081 28714750

[B27] Hendricks S. Hill H. Hollander S. Lombard W. Parker R. (2020). Effects of foam rolling on performance and recovery: a systematic review of the literature to guide practitioners on the use of foam rolling. J. Bodyw. Mov. Ther. 24 (2), 151–174. 10.1016/j.jbmt.2019.10.019 32507141

[B28] Hsia J. Zhao G. Town M. (2020). Estimating undercoverage bias of internet users. Prev. Chronic Dis. 17, E104. 10.5888/PCD17.200026 32915129 PMC7553209

[B29] Hughes G. A. Ramer L. M. (2019). Duration of myofascial rolling for optimal recovery, range of motion, and performance: a systematic review of the literature. Int. J. Sports Phys. Ther. 14 (6), 845–859. 10.26603/ijspt20190845 31803517 PMC6878859

[B30] Kasahara K. Konrad A. Yoshida R. Murakami Y. Sato S. Aizawa K. (2022). Comparison between 6-week foam rolling intervention program with and without vibration on rolling and non-rolling sides. Eur. J. Appl. Physiology 122 (9), 2061–2070. 10.1007/S00421-022-04975-7 35704122

[B31] Kasahara K. Konrad A. Yoshida R. Murakami Y. Sato S. Koizumi R. (2024). Comparison of acute and prolonged effects of short-term foam rolling and vibration foam rolling on the properties of knee extensors. Biol. Sport 41 (2), 19–26. 10.5114/BIOLSPORT.2024.129488 38524825 PMC10955736

[B32] Kent M. L. Tighe P. J. Belfer I. Brennan T. J. Bruehl S. Brummett C. M. (2017). The ACTTION–APS–AAPM pain taxonomy (AAAPT) multidimensional approach to classifying acute pain conditions. Pain Med. 18 (5), 479–489. 10.1016/j.jpain.2017.02.421 28495013 PMC7323793

[B33] Konrad A. Nakamura M. Bernsteiner D. Tilp M. (2021). The accumulated effects of foam rolling combined with stretching on range of motion and physical performance: a systematic review and meta-analysis. J. Sports Sci. and Med. 20 (3), 535–545. 10.52082/jssm.2021.535 34267594 PMC8256518

[B34] Konrad A. Nakamura M. Behm D. G. (2022). The effects of foam rolling training on performance parameters: a systematic review and meta-analysis including controlled and randomized controlled trials. Int. J. Environ. Res. Public Health 19 (18), 11638. 10.3390/IJERPH191811638 36141907 PMC9517147

[B35] Loeser J. D. Melzack R. (1999). Pain: an overview. Lancet 353 (9164), 1607–1609. 10.1016/S0140-6736(99)01311-2 10334273

[B36] Martínez-Aranda L. M. Sanz-Matesanz M. García-Mantilla E. D. González-Fernández F. T. (2024). Effects of self-myofascial release on athletes’ physical performance: a systematic review. J. Funct. Morphol. Kinesiol. 9 (1), 20. 10.3390/JFMK9010020 38249097 PMC10801590

[B37] Michalak B. Kopiczko A. Gajda R. Adamczyk J. G. (2024). Recovery effect of self-myofascial release treatment using different type of a foam rollers. Sci. Rep. 14 (1), 15762. 10.1038/S41598-024-66577-X 38982124 PMC11233653

[B38] Nakamura M. Sato S. Kiyono R. Yoshida R. Yasaka K. Yahata K. (2022). Comparison between foam rolling with and without vibration on passive and active plantar Flexor muscle properties. J. Strength Cond. Res. 36 (12), 3339–3344. 10.1519/JSC.0000000000004123 34474432 PMC7613848

[B39] Okamoto T. Masuhara M. Ikuta K. (2014). Acute effects of self-myofascial release using a foam roller on arterial function. J. Strength Cond. Res. 28 (1), 69–73. 10.1519/JSC.0B013E31829480F5 23575360

[B40] Pagaduan J. C. Chang S. Y. Chang N. J. (2022). Chronic effects of foam rolling on flexibility and performance: a systematic review of randomized controlled trials. Int. J. Environ. Res. Public Health 19 (7), 4315. 10.3390/IJERPH19074315 35409995 PMC8998857

[B41] Park S. J. Lee S. I. Jeong H. J. Kim B. G. (2021). Effect of vibration foam rolling on the range of motion in healthy adults: a systematic review and meta-analysis. J. Exerc. Rehabilitation 17 (4), 226–233. 10.12965/JER.2142322.161 34527633 PMC8413912

[B42] Perna G. Nemeroff C. B. (2021). Can personalized medicine mitigate confirmation bias in mental health? Braz. J. Psychiatry 44 (2), 121–123. 10.1590/1516-4446-2021-0032 34669842 PMC9041965

[B43] Prince S. A. Lang J. J. de Groh M. Badland H. Barnett A. Littlejohns L. B. (2023). Prioritizing a research agenda on built environments and physical activity: a twin panel Delphi consensus process with researchers and knowledge users. Int. J. Behav. Nutr. Phys. Activity 20 (1), 144. 10.1186/S12966-023-01533-Y 38062460 PMC10704660

[B44] Rabe E. Partsch H. Morrison N. Meissner M. H. Mosti G. Lattimer C. R. (2020). Risks and contraindications of medical compression treatment - a critical reappraisal. An international consensus statement. Phlebology 35 (7), 447–460. 10.1177/0268355520909066 32122269 PMC7383414

[B45] Skinner B. Moss R. Hammond L. (2020). A systematic review and meta-analysis of the effects of foam rolling on range of motion, recovery and markers of athletic performance. J. Bodyw. Mov. Ther. 24 (3), 105–122. 10.1016/j.jbmt.2020.01.007 32825976

[B46] Stone A. A. Schneider S. Smyth J. M. Junghaenel D. U. Couper M. P. Wen C. (2024). A population-based investigation of participation rate and self-selection bias in momentary data capture and survey studies. Curr. Psychol. 43 (3), 2074–2090. 10.1007/S12144-023-04426-2/TABLES/5 PMC12456455 40994465

[B47] Vural F. Ciftci S. Vural B. (2015). The knowledge, attitude and behaviours of nurses about pharmacovigilance, adverse drug reaction and adverse event reporting in a state hospital. North. Clin. Istanbul 1 (3), 147–152. 10.14744/NCI.2014.41636 28058321 PMC5175033

[B48] Warneke K. Konrad A. Wilke J. (2024). The knowledge of movement experts about stretching effects: does the science reach practice? PloS One 19 (1), e0295571. 10.1371/JOURNAL.PONE.0295571 38277378 PMC10817148

[B49] Wiewelhove T. (2021). Rollen oder Nichtrollen: evidenz der Wirksamkeit von Foam-Rolling. B&G Bewegungstherapie Und Gesundheitssport 37 (02), 60–65. 10.1055/A-1380-9413

[B50] Wiewelhove T. Döweling A. Schneider C. Hottenrott L. Meyer T. Kellmann M. (2019). A meta-analysis of the effects of foam rolling on performance and recovery. Front. physiology 10, 376. 10.3389/fphys.2019.00376 31024339 PMC6465761

[B51] Wilke J. Müller A.-L. Giesche F. Power G. Ahmedi H. Behm D. G. (2020). Acute effects of foam rolling on range of motion in healthy adults: a systematic review with multilevel meta-analysis. Sports Med. Auckl. N.Z. 50 (2), 387–402. 10.1007/s40279-019-01205-7 31628662

[B52] Zidarov D. Thomas A. Poissant L. (2013). Knowledge translation in physical therapy: from theory to practice. Disabil. Rehabilitation 35 (18), 1571–1577. 10.3109/09638288.2012.748841 23339718

